# Influence of subsidence after stand-alone anterior cervical discectomy and fusion in patients with degenerative cervical disease: A long-term follow-up study

**DOI:** 10.1097/MD.0000000000030673

**Published:** 2022-09-23

**Authors:** Han-Seung Ryu, Moon-Soo Han, Shin-Seok Lee, Bong Ju Moon, Jung-Kil Lee

**Affiliations:** a Department of Neurosurgery, Chonnam National University Medical School & Research Institute of Medical Sciences, Gwangju, Korea; b Division of Rheumatology, Department of Internal Medicine, Chonnam National University Hospital and Medical School, Gwangju, South Korea.

**Keywords:** anterior cervical discectomy and fusion, cervical alignment, long-term, stand-alone, subsidence

## Abstract

This study aimed to evaluate the influence of subsidence in patients who performed stand-alone anterior cervical discectomy and fusion (ACDF) by analyzing the long-term clinical and radiological outcomes. This retrospective study enrolled 53 patients with 79 segments with degenerative cervical disease treated with stand-alone ACDF with ≥5 years of follow-up. Segmental angle (SA), cervical sagittal alignment (CSA), subsidence, and fusion were analyzed. Visual analog scale (VAS) scores and neck disability index (NDI) were also evaluated. Subsidence occurred in 24 (45.2%) patients and 38 segments (48.1%) at the last follow-up. The mean VAS score and NDI had improved in both the subsidence and non- subsidence groups. The mean SA at the last follow-up had increased to 1.3° ± 8.5° in the subsidence group and to 1.5° ± 5.2° in the non-subsidence group compared with the post-operative SA (*P* < .001). The overall mean CSA at the last follow-up increased over time in both the groups compared with the post-operative CSA (*P* = .003). The fusion rate at 1 year after surgery was 86.8% and 82.9% in the subsidence and non-subsidence groups, respectively. However, the differences in the SA, CSA, and fusion rates between the groups were not statistically significant (*P* = .117, .98, and .682, respectively). Subsidence after stand-alone ACDF occurs to a certain capacity; however, it does not appear to significantly influence the radiological and clinical outcomes if foramen decompression is adequately and sufficiently provided in a long-term follow-up study. In contrast, subsidence appears to positively affect the fusion rate in the short-term follow-up.

## 1. Introduction

Anterior cervical discectomy and fusion (ACDF) with plate fixation has been recommended for degenerative cervical disease involving two or more levels, with instability and kyphosis.^[1,2]^ However, addition of a cervical plate is associated with cost issues and plate-related complications, such as screw failure and dysphagia.^[3–5]^ Therefore, several studies suggest that stand-alone ACDF without plate fixation is an acceptable surgical option for cervical degenerative disease.^[6,7]^ However, the majority of these reports had a follow-up period of ≤2 years. Meanwhile, the most critical issue of stand-alone ACDF is subsidence, which can cause cervical kyphotic changes or clinical deterioration. According to some studies, cage subsidence occurred in 19% to 63% of the patients after stand-alone ACDF.^[8–12]^ Moreover, some studies have demonstrated that stand-alone ACDF was unsatisfactory, with worse radiological outcomes noted due to subsidence, compared with standard ACDF with plate fixation.^[13,14]^ In contrast, some studies have reported that cage subsidence was not significantly associated with clinical and radiological outcomes.^[15,16]^

This study aimed to evaluate the influence of subsidence in patients who underwent stand-alone ACDF using polyether ether ketone (PEEK) cages without plate fixation by analyzing the long-term clinical and radiological outcomes.

## 2. Materials and Methods

### 2.1. Patient population and study design

This study was performed according to the requirements associated with patient anonymity and was approved by the Institutional Review Board (CNUH-2020-054).

We conducted a retrospective review of 202 patients with degenerative cervical disease who performed ACDF with or without plate fixation performed by a single neurosurgeon between 2009 and October 2015 at our institution. The inclusion criteria were as follows: patients with cervical radiculopathy or cervical spondylotic myelopathy unresponsive to medical treatment; patients treated with one-, two-, or multiple-level stand-alone ACDF; patients with a clinical and radiological follow-up of ≥60 months after surgery. The exclusion criteria were as follows: history of cervical spine surgery; presence of other cervical diseases, including infections or tumors; patients with clinical or radiological follow-up of <60 months after surgery.

In total, we enrolled 53 study patients with degenerative cervical disease who performed stand-alone ACDF with a follow-up duration of ≥60 months. To identify the patients’ clinical characteristics, the clinical data at the time of treatment (including age, sex, presenting symptoms, bone mineral density, level of surgery, plain radiographs, computed tomography [CT] findings, and magnetic resonance imaging findings of the cervical spine) were collected.

### 2.2. Surgical technique

General anesthesia was used for all the patients. The standard Smith–Robinson method was used to expose the involved segment. After exposing the affected prevertebral space, the alignment and affected levels were confirmed by intraoperative fluoroscopy. After discectomy, the posterior annulus, posterior longitudinal ligament, and osteophytes were removed. In most cases, uncoforaminotomy was sufficiently performed to totally decompress the nerve roots. The upper and lower cartilaginous endplates were decorticated, preserving the bony endplates. Local autologous bone chips were collected during removal of osteophytes for grafting. The appropriate PEEK cage was selected (Solis cage; Stryker, Allendale, NJ/Cornerstone cage; Medtronic, Memphis, TN), and the cages were filled with a demineralized bone matrix (Surefuse^R^; Hansbiomed Corp, Seoul, South Korea), intermixed with autologous bone chips, and inserted into the disc space. The operation was completed without any anterior plating. All the patients wore a Philadelphia cervical collar (Philadelphia Cervical Collar Co., Thorofare, NJ) for 8 weeks.

### 2.3. Radiological and clinical evaluation

**Figure 1.**
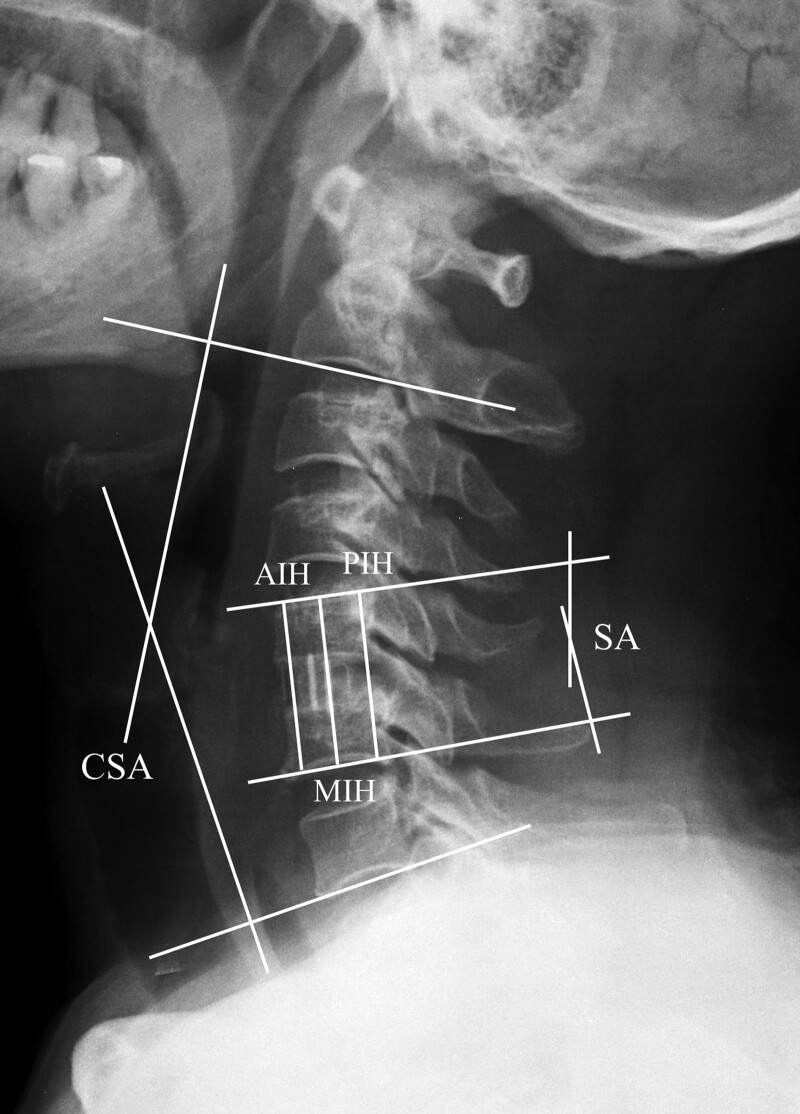
Cervical sagittal angle (CSA), segmental angle (SA), and anterior, middle, and posterior intervertebral height (AIH, MIH, and PIH) on lateral radiographs of the cervical spine.

Plain radiographs were obtained in the anteroposterior, lateral, flexion, and extension views pre-operatively, immediately after surgery, 6 months after surgery, and subsequently once a year. Moreover, pre-operative cervical spine CT and magnetic resonance imaging images were obtained. Follow-up CT was performed at 6 months after surgery and subsequently once a year to evaluate fusion. Fusion was defined as motion of <2 mm between the spinous processes on flexion-extension lateral radiographs, <50% of radiolucency covering the implant’s outer surface, and the presence of continuous bridging trabeculae at the graft on simple lateral radiographs or CT images.^[17]^ Segmental angle (SA), cervical sagittal alignment (CSA), and subsidence were measured using lateral plain radiographs. The SA was measured using the Cobb angle, that is, the angle between the superior endplate of the upper adjacent vertebra and the inferior endplate of the lower adjacent vertebra at the operated disc level. The overall CSA was measured according to the Cobb angle between the lower endplate of C2 and lower endplate of C7. Kyphosis was noted as a positive value, whereas lordosis was noted as a negative value. The total intervertebral height (TIH) was measured at the anterior, middle, and posterior points of the superior endplate of the upper adjacent vertebra and the inferior endplate of the lower adjacent vertebra at the operated disc level (anterior intervertebral height [AIH], middle intervertebral height [MIH], and posterior intervertebral height [PIH]) (Fig. 1). Based on a serial TIH analysis, subsidence was defined as a decrease in the TIH of >3.0 mm at any three points compared with the recorded post-operative TIH.^[10,13,18]^ Two independent surgeons evaluated all the radiographs and CT images.

To evaluate pain in the neck and arms, a visual analog scale (VAS) was used pre- and post-operatively. The neck disability index (NDI) questionnaire was administered pre- and post-operatively and at the last follow-up.

### 2.4. Statistical analysis

We analyzed the radiological and clinical outcomes between the subsidence and non-subsidence groups. Data were entered into SPSS version 25.0 (SPSS, IBM, Chicago, IL). The chi-square test was used to compare qualitative data between the groups. One way analysis of variance was used for quantitative data. A paired samples *t* test was used to evaluate the results pre- and post-operatively. The independent *t* test and Mann–Whitney *U* test for parametric variables were used for analyzing relationships between the clinical and radiological outcomes and subsidence. Quantitative data are presented as mean and standard deviation and qualitative data as frequency or percentage. A *P* value of <.05 was considered to be statistically significant.

## 3. Results

### 3.1. Patient characteristics

This study examined 79 treated segments in 53 patients. The mean age of the patients was 60 (range, 33–75) years at the time of the stand-alone ACDF. There were 36 (67.9%) male and 17 (32.1%) female patients. Further, 29 (54.7%), 22 (41.6%), and 2 (3.7%) patients underwent stand-alone ACDF surgery at one, two, and three segments, respectively. Stand-alone ACDF was performed more at the C4–5 level (34 segments, 43.1%), followed by the C5–6 (25 segments, 31.7%), C6–7 (14 segments, 17.6%), and C3–4 (6 segments, 7.6%) levels. The mean follow-up period after surgery was 89 (range, 60–162) months. The baseline and pre-operative clinical characteristics of the enrolled patients are presented in Table 1.

**Table 1:** Clinical characteristics of patients.

Number of patients	53
Mean age; yr (range)	60 (33–75)
Sex, n (%)	
Male	36 (67.9%)
Female	17 (32.1%)
BMD; mean	−1.5 ± 0.8
Stand-alone ACDF	
One segment	29 (54.7%)
Two segments	22 (41.6%)
Three segments	2 (3.7%)
Level of segments, n (%)	79
C3–4	6 (7.6%)
C4–5	34 (43.1%)
C5–6	25 (31.7%)
C6–7	14 (17.6%)
Pre-operative TIH; mean (mm)	
AIH	35.2 ± 3.8
MIH	34.4 ± 3.4
PIH	34.7 ± 3.4
Pre-operative SA; mean (^o^)	−2.1 ± 6.6
Pre-operative CSA; mean (^o^)	−10.2 ± 15.2
Pre-operative VAS neck	6.4 ± 2.1
Pre-operative VAS arm	6.8 ± 1.3
Pre-operative NDI	20.7 ± 10.5
Fusion rate	
1-yr after surgery	67 (84.8%)
Last follow-up	79 (100%)
Mean follow-up period; mo (range)	89 (60–162)

ACDF = anterior cervical discectomy and fusion, AIH = anterior intervertebral height, BMD = bone mineral density, C = cervical, CSA = cervical sagittal angle, MIH = middle intervertebral height, NDI = neck disability index, PIH = posterior intervertebral height, SA = segmental angle, TIH = total intervertebral height, VAS = visual analog scale.

### 3.2. Radiological outcomes

**Figure 2.**
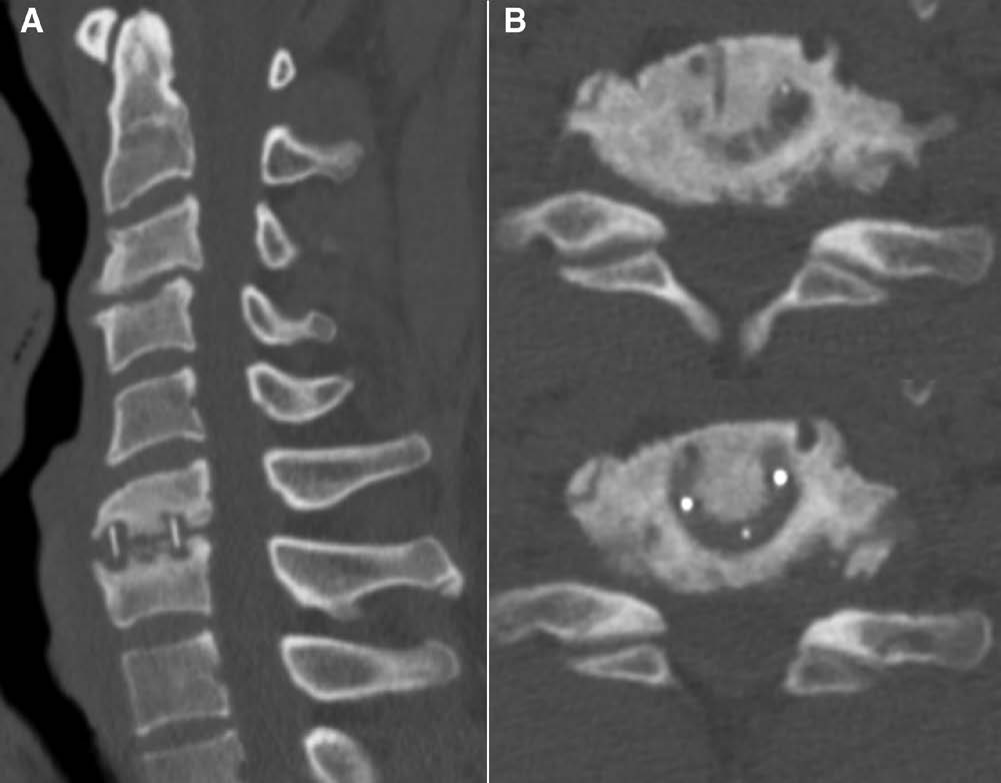
(A) Post-operative computed tomography (CT) image obtained 10 months after surgery showing subsidence at the C6–7 level. (B) Axial CT image at the C6–7 level showing that right-side foraminal stenosis occurred alongside subsidence when uncoforaminotomy was insufficiently performed.

Fusion was achieved for 67 segments (84.8%) at 1 year post-operatively and for 79 segments (100%) at the last follow-up. Additional surgical treatments for fusion were not required during the follow-up period. Subsidence occurred in 38 segments (48.1%) in 24 (45.2%) patients at the last follow-up. We divided the patients into the subsidence and non-subsidence groups for a comparative analysis. There were 18 men and six women with a mean age of 54.3 (range, 31–75) years in the subsidence group and 18 men and 11 women with a mean age of 50.9 (range, 33–71) years in the non-subsidence group. The mean follow-up period was 75.5 (range, 60–108) months and 83.8 (range, 60–162) months in the subsidence and non-subsidence groups, respectively. No significant difference was noted in the number of segments involved (single- vs multi-level) or the cage material used (Solis cage vs. Cornerstone cage) between the two groups (Table 2). The mean AIH, middle MIH, and PIH reported at the last follow-up decreased by 5.5 ± 1.5, 3.4 ± 1.4, and 3.6 ± 1.5 mm, respectively, in the subsidence group and by 1.6 ± 1.4, 1.5 ± 1.2, and 1.2 ± 1.8 mm, respectively, in the non-subsidence group compared with the values recorded immediately after surgery. Although the AIH, MIH, and PIH decreased significantly from after the surgery to the last follow-up in both the groups, the TIH decreased significantly more in the subsidence group than in the non-subsidence group (*P* < .001) (Table 3). The AIH, MIH, and PIH had mainly changed within 1 year after surgery (Fig. 2). The mean SA at the last follow-up increased significantly by 1.3° ± 8.5° at the last follow-up in the subsidence group and by 1.5° ± 5.2° in the non-subsidence group compared with the post-operative values (*P* < .001). The mean CSA at the last follow-up increased significantly in both the groups compared with the values recorded post-operatively (*P* = .003). However, the mean SA and CSA difference between the groups were not statistically significant (*P* = .117 and *P* = .98, respectively). The fusion rate at 1 year after surgery was 86.8% in the subsidence group (33 of 38 segments) and 82.9% in the non-subsidence group (34 of 41 segments) on segmental analysis, and the difference in the fusion rates between the two groups was not statistically significant (*P* = .682) (Table 3).

**Table 2:** Characteristics of patients in the subsidence and non-subsidence groups.

	Subsidence group	Non-subsidence group	*P* value^*^
Number of patients	24	29	
Mean age, yr	54.3	50.9	.104
Sex ratio (male:female)	18:6	18:11	.051
BMD	−1.43 ± 1.0	−1.52 ± 0.8	.793
Follow-up period (mo)	75.5	83.8	.078
Level of segments, n (%)	38 (48.1%)	41 (51.9%)	.847
C3–4	4 (10.5%)	2 (4.9%)	
C4–5	16 (42.1%)	18 (43.9%)	
C5–6	12 (31.6%)	13 (31.7%)	
C6–7	6 (15.8%)	8 (19.5%)	
Number of segments, n (%)			
Single-level	11 (37.9%)	18 (62.1%)	.057
Multi-level (two or three)	13 (54.2%)	11 (45.8%)	.803
Cage material, n (%)			
Solis	20 (44.4%)	25 (55.6%)	.819
Cornerstone	18 (52.9%)	16 (47.1%)	.841

BMD = bone mineral density, C = cervical.

* Comparison of mean values between the subsidence and non-subsidence groups.

**Table 3:** Radiological outcomes in the subsidence and non-subsidence groups.

	Subsidence group	Non-subsidence group	*P* value^‡^
AIH (mm)			<.001*
Pre-operation	35.7 ± 3.1	34.7 ± 4.6	.476^†^
Post-operation	36.8 ± 5.1	35.2 ± 3.7	.152^†^
1-yr follow-up	32.9 ± 4.4	33.9 ± 1.9	.499^†^
Last follow-up	31.3 ± 3.6	33.7 ± 3.3	.186^†^
Δ Post-op–last f/u	5.5 ± 1.5	1.6 ± 1.4	<.001^†^
MIH (mm)			<.001*
Pre-operation	34.5 ± 2.5	34.3 ± 4.2	.831^†^
Post-operation	36.5 ± 4.5	35.4 ± 3.5	.155^†^
1-yr follow-up	32.9 ± 3.9	34.4 ± 1.7	.261^†^
Last follow-up	32.6 ± 3.1	33.9 ± 3.9	.110^†^
Δ Post-op–last f/u	3.4 ± 1.4	1.5 ± 1.2	<.001^†^
PIH (mm)			<.001*
Pre-operation	34.9 ± 2.4	34.5 ± 4.4	.812^†^
Post-operation	36.5 ± 4.3	35.3 ± 3.2	.112^†^
1-yr follow-up	33.2 ± 3.8	34.9 ± 2.1	.353^†^
Last follow-up	32.9 ± 2.8	34.1 ± 4.0	.399^†^
Δ Post-op–last f/u	3.6 ± 1.5	1.2 ± 1.8	<.001^†^
Segmental angle (^o^)			<.001*
Pre-operation	−1.7 ± 7.3	−2.5 ± 6.0	.190^†^
Post-operation	−2.4 ± 5.9	−3.8 ± 4.7	.737^†^
1-yr follow-up	−1.9 ± 7.4	−2.6 ± 5.1	.668^†^
Last follow-up	−0.9 ± 8.0	−2.3 ± 5.0	.301^†^
Δ Last f/u–post-op	1.3 ± 8.5	1.5 ± 5.2	.117^†^
Cervical sagittal angle (^o^)			.003*
Pre-operation	−8.2 ± 18.3	−12.2 ± 13.0	.385^†^
Post-operation	−10.5 ± 10.2	−14.6 ± 7.9	.094^†^
1-yr follow-up	−8.1 ± 13.3	−12.8 ± 7.4	.887^†^
Last follow-up	−7.7 ± 11.9	−12.2 ± 6.4	.884^†^
Δ Last f/u–post-op	3.8 ± 9.8	2.4 ± 7.8	.098^†^
Fusion rate			
1-yr after surgery	33 (86.8%)	34 (82.9%)	.682^†^
Last follow-up	38 (100%)	41 (100%)	

AIH = anterior intervertebral height, f/u = follow-up, MIH = middle intervertebral height, PIH = posterior intervertebral height, post-op = postoperatively.

* Comparison of mean values between post-operation and last follow-up.

† Comparison of mean values between the subsidence and non-subsidence groups.

‡ Paired *t* test was used for analysis.

### 3.3. Clinical outcomes

The overall pre-operative VAS score for neck pain was 6.4 ± 2.1 and 3.1 ± 1.7 at the last follow-up. When comparing the subsidence and non-subsidence groups, the score had significantly improved to 3.2 ± 2.2 and 3.7 ± 1.4 in the subsidence and non-subsidence groups, respectively (*P* < .001) at the last follow-up, and no significant difference was noted between the groups (*P* = .441). The overall pre-operative VAS score for arm pain was 6.8 ± 1.3 and 3.5 ± 1.8 at the last follow-up. Similarly, the VAS score had improved significantly to 3.2 ± 1.1 and 3.5 ± 1.3 in the subsidence and non-subsidence groups, respectively (*P* < .001) at the last follow-up, with no significant difference between the groups (*P* = .631). Furthermore, the NDI had improved over time during the follow-up period in both the groups (*P* < .001), but the difference was not statistically significant (*P* = .705) (Table 4). In one patient in the subsidence group during the follow-up period, the VAS score for neck pain had improved; however, the score for arm pain had deteriorated 10 months after surgery. Moreover, posterior cervical foraminotomy was performed to manage radiating arm pain (Fig. 2). After posterior cervical foraminotomy, the VAS score of the patient for arm pain had improved.

**Table 4:** Clinical outcomes in the subsidence and non-subsidence groups.

	Subsidence group	Non-subsidence group	*P* value^‡^
VAS_neck			<.001^*^
Pre-operation	6.3 ± 2.6	6.8 ± 1.5	.226^†^
Last follow-up	3.1 ± 2.2	3.0 ± 1.0	.831^†^
ΔPre-op–last f/u	3.2 ± 2.2	3.7 ± 1.4	.441^†^
VAS_arm			<.001^*^
Pre-operation	6.8 ± 1.1	6.8 ± 1.5	.916^†^
Last follow up	3.9 ± 1.8	3.3 ± 1.8	.350^†^
ΔPre-op–last f/u	3.2 ± 1.1	3.5 ± 1.3	.631^†^
NDI			<.001^*^
Pre-operation	19.3 ± 9.8	22.4 ± 11.6	.440^†^
Last follow-up	4.5 ± 3.4	6.3 ± 5.9	.336^†^
ΔPre-op–last f/u	13.8 ± 10.1	15.7 ± 14.7	.705^†^

f/u = follow-up, NDI = neck disability index, post-op = post-operation, pre-op = pre-operation, VAS = visual analog scale.

* Comparison of mean values between the post-operation and last follow-up.

† Comparison of mean values between the subsidence and non-subsidence groups.

‡ Paired *t* test was used for analysis.

## 4. Discussion

ACDF with plate fixation is considered as the “gold standard” for stable cervical interbody fusion in treating degenerative cervical disease.^[1,2]^ Anterior plate fixation improves the cervical spine stability, maintains the intervertebral height, and enhances the fusion rate. Consequently, it reduces complications such as post-operative graft collapse and loss of cervical physiological curvature.^[19]^ In addition, various materials have been developed for interbody grafts with ACDF to avoid the morbidity associated with autologous bone grafts. In particular, PEEK cages have been developed to acquire immediate stability and successful bone fusion.^[5]^ The development of bone-inductive substances and synthetic cages increases and accelerates the fusion rate, raising questions about the need for additional anterior plate fixation, leading to complications such as material failure and dysphagia.^[3,4,20,21]^ Recently, several studies have suggested that stand-alone ACDF is a secure and effective treatment modality for cervical degenerative disease.^[6,7,22–24]^ However, this proposal remains controversial because these reports included a follow-up period of only ≤2 years. The present study analyzed the long-term radiological and clinical outcomes with a follow-up period of >5 years after stand-alone ACDF.

**Figure 3.**
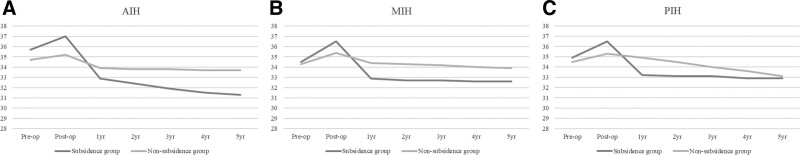
Changes in the (A) anterior, (B) middle, and (C) posterior intervertebral height (AIH, MIH, and PIH) between the subsidence and non-subsidence groups recorded pre-operatively, immediately after surgery, at 1 and 2 years post-operatively, and at the last follow-up.

A significant issue associated with stand-alone ACDFs is subsidence. The generally accepted hypothesis about subsidence after stand-alone ACDF is that the intervertebral space collapses as the implant penetrates the vertebral bodies, given that there is no plate to support axial loading. Kim et al^[13]^ reported that subsidence occurred in 58.6% of the patients after stand-alone ACDF with an average follow-up of 5 years (range, 28–135 months); they suggested that the subsidence progressed over time continued until the last follow-up. In addition, the VAS scores for neck and arm increased. When patient satisfaction was assessed at the last follow-up using Odom’s criteria, 64.6% of the patients selected “unsatisfactory” response. However, a significant association was not found between subsidence and the clinical outcomes; there was no long-term follow-up in all the patients, and the surgeries were not performed by a single surgeon in their study. Several previous reports have demonstrated that the subsidence did not significantly influence the clinical outcomes, and that subsidence mostly occurred within 1 year after surgery and stabilized subsequently.^[14–16,25]^ In our study, although the prevalence of subsidence was 48.1%, neck and arm pain had reduced, and the NDI had improved at the last follow-up (*P* < .001). Although radiating arm pain in one patient in the subsidence group recurred, it improved after posterior cervical foraminotomy, and the incidence rate was not high (2.6%, 1 of 38 segments with subsidence). It may be assumed that the foraminal height also decreases with subsidence, thereby affecting the clinical outcome. Therefore, we suggest that subsidence does not influence the clinical outcome if foramen decompression is performed adequately and sufficiently during surgery. In addition, our follow-up of 5 years supported the findings of previous reports that a decrease in the intervertebral height mostly occurred within 1 year after surgery; thereafter, the tendency of decrease in the intervertebral height was negligible until the last follow-up (Fig. 3).

To date, several studies have reported that stand-alone ACDF acquired a successful fusion rate of >90%.^[7,22,23]^ In our study, the fusion rate was 84.8% at 1 year after surgery and 100% at the last follow-up with good stability. With the development of bone-inductive substances and synthetic cages, additional anterior plate fixation in ACDF does not seem to offer high fusion rates. Meanwhile, Wu et al^[11]^ reported that anterior plate fixation may act as a shield for the mechanical axial load, which is critical for the fusion process. Their study hypothesized that during the bone fusion process, cage subsidence contributes to mechanical axial loading of the bone graft inside the cage, thus facilitating fusion.^[8,12]^ In our study, the fusion rate was higher in the subsidence group than in the non-subsidence group 1 year after surgery, although not at a statistically significant level (*P* = .682). Regarding cervical stability, although the changes in the SA and CSA from the post-operative period to the last follow-up were larger in the subsidence group than in the non-subsidence group, the difference between the groups was not statistically significant. Thus, it can be assumed that the pre-operative SA and CSA may affect the changes in the post-operative SA and CSA. Consequently, stand-alone ACDF without anterior plate fixation does not appear to be the only factor involved in the deterioration of the SA and CSA.

Except for subsidence, no other surgical complications were reported, such as those related to the cage, bone-inductive substances, or the anterior plate and screw. Additional surgical treatment for cervical fusion was not necessary throughout the follow-up period. Most previous reports have failed to identify subsidence as a factor predicting low fusion rates, kyphotic change, and unsatisfactory clinical outcomes after surgery.^[15,16]^ Subsidence is an unavoidable consequence of stand-alone ACDF; if foramen decompression is adequately and sufficiently performed, proper subsidence with cages settlement into the vertebral bodies may contribute to the bone fusion process and not adversely affect the clinical outcomes and cervical alignment.

This retrospective study had some limitations. This study included a small number of patients, and there was no control group for comparison of the radiological and clinical outcomes. The enrolled patients in this study were not randomized meaning biased data. Moreover, this study did not identify the factors predicting subsidence and kyphotic changes. Further randomized prospective studies are required to determine the efficacy and toxicity of stand-alone ACDF surgery. In addition, research is needed to identify predicting factors that can cause subsidence and kyphotic changes considering gender and bone mineral density.

## 5. Conclusion

Stand-alone ACDF does not necessarily provide better outcomes than standard techniques, such as ACDF with plate fixation. Subsidence after stand-alone ACDF occurs to a certain capacity me capacity; however, it does not appear to significantly influence the radiological and clinical outcomes if foramen decompression is adequately and sufficiently provided, as shown in our long-term follow-up study. In contrast, subsidence appears to positively affect the fusion rate in the short-term follow-up.

## Author contributions

**Conceptualization:** Moon-Soo Han, Jung-Kil Lee.

**Data curation:** Han-Seung Ryu, Moon-Soo Han, Shin-Seok Lee, Bong Ju Moon, Jung-Kil Lee.

**Formal analysis:** Han-Seung Ryu, Moon-Soo Han, Jung-Kil Lee.

**Funding acquisition:** Jung-Kil Lee.

**Investigation:** Jung-Kil Lee.

**Project administration:** Jung-Kil Lee.

**Supervision:** Moon-Soo Han, Shin-Seok Lee, Bong Ju Moon, Jung-Kil Lee.

**Writing – original draft:** Han-Seung Ryu.

**Writing – review & editing:** Moon-Soo Han, Jung-Kil Lee.

## References

[1] Papacci F Rigante L Fernandez E. Anterior cervical discectomy and interbody fusion with porous tantalum implant. Results in a series with long-term follow-up. J Clin Neurosci. 2016;33:159–62. 27452131 10.1016/j.jocn.2016.03.036

[2] Yue WM Brodner W Highland TR. Long-term results after anterior cervical discectomy and fusion with allograft and plating: a 5- to 11-year radiologic and clinical follow-up study. Spine (Phila Pa 1976). 2005;30:2138–44. 16205338 10.1097/01.brs.0000180479.63092.17

[3] Marawar S Girardi FP Sama AA. National trends in anterior cervical fusion procedures. Spine (Phila Pa 1976). 2010;35:1454–9. 20216341 10.1097/BRS.0b013e3181bef3cb

[4] Schroder J Grosse-Dresselhaus F. PMMA versus titanium cage after anterior cervical discectomy - a prospective randomized trial. Zentralbl Neurochir. 2007;68:2–7. 16969747 10.1055/s-2006-942184

[5] Cho DY Liau WR Lee WY. Preliminary experience using a polyetheretherketone (PEEK) cage in the treatment of cervical disc disease. Neurosurgery. 2002;51: 1343–9; discussion 1349-1350. 49; discussion 1349 12445338

[6] Pereira EA Chari A Hempenstall J. Anterior cervical discectomy plus intervertebral polyetheretherketone cage fusion over three and four levels without plating is safe and effective long-term. J Clin Neurosci. 2013;20:1250–5. 23890411 10.1016/j.jocn.2012.10.028

[7] Shiban E Gapon K Wostrack M. Clinical and radiological outcome after anterior cervical discectomy and fusion with stand-alone empty polyetheretherketone (PEEK) cages. Acta Neurochir (Wien). 2016;158:349–55. 26620448 10.1007/s00701-015-2630-2

[8] Bartels RH Donk RD Feuth T. Subsidence of stand-alone cervical carbon fiber cages. Neurosurgery. 2006;58:502–8; discussion 502-508. 16528190 10.1227/01.NEU.0000197258.30821.50

[9] Barsa P Suchomel P. Factors affecting sagittal malalignment due to cage subsidence in standalone cage assisted anterior cervical fusion. Eur Spine J. 2007;16:1395–400. 17221174 10.1007/s00586-006-0284-8 PMC2200763

[10] Gercek E Arlet V Delisle J. Subsidence of stand-alone cervical cages in anterior interbody fusion: warning. Eur Spine J. 2003;12:513–6. 12827473 10.1007/s00586-003-0539-6 PMC3468003

[11] Wu WJ Jiang LS Liang Y. Cage subsidence does not, but cervical lordosis improvement does affect the long-term results of anterior cervical fusion with stand-alone cage for degenerative cervical disc disease: a retrospective study. Eur Spine J. 2012;21:1374–82. 22205113 10.1007/s00586-011-2131-9 PMC3389116

[12] Tome-Bermejo F Morales-Valencia JA Moreno-Perez J. Degenerative cervical disc disease: long-term changes in sagittal alignment and their clinical implications after cervical interbody fusion cage subsidence: a prospective study with standalone lordotic tantalum cages. Clin Spine Surg. 2017;30:E648–55. 28525492 10.1097/BSD.0000000000000293

[13] Kim WB Hyun SJ Choi H. Long-term follow-up results of anterior cervical inter-body fusion with stand-alone cages. J Korean Neurosurg Soc. 2016;59:385–91. 27446521 10.3340/jkns.2016.59.4.385 PMC4954888

[14] Lee CH Hyun SJ Kim MJ. Comparative analysis of 3 different construct systems for single-level anterior cervical discectomy and fusion: stand-alone cage, iliac graft plus plate augmentation, and cage plus plating. J Spinal Disord Tech. 2013;26:112–8. 23027363 10.1097/BSD.0b013e318274148e

[15] Karikari IO Jain D Owens TR. Impact of subsidence on clinical outcomes and radiographic fusion rates in anterior cervical discectomy and fusion: a systematic review. J Spinal Disord Tech. 2014;27:1–10. 24441059 10.1097/BSD.0b013e31825bd26d

[16] Joo YH Lee JW Kwon KY. Comparison of fusion with cage alone and plate instrumentation in two-level cervical degenerative disease. J Korean Neurosurg Soc. 2010;48:342–6. 21113362 10.3340/jkns.2010.48.4.342 PMC2982913

[17] Thome C Krauss JK Zevgaridis D. A prospective clinical comparison of rectangular titanium cages and iliac crest autografts in anterior cervical discectomy and fusion. Neurosurg Rev. 2004;27:34–41. 12905078 10.1007/s10143-003-0297-2

[18] Haden N Latimer M Seeley HM. Loss of inter-vertebral disc height after anterior cervical discectomy. Br J Neurosurg. 2005;19:469–74. 16574558 10.1080/02688690500495109

[19] Li J Zheng Q Guo X. Anterior surgical options for the treatment of cervical spondylotic myelopathy in a long-term follow-up study. Arch Orthop Trauma Surg. 2013;133:745–51. 23503888 10.1007/s00402-013-1719-4

[20] Moreland DB Asch HL Clabeaux DE. Anterior cervical discectomy and fusion with implantable titanium cage: initial impressions, patient outcomes and comparison to fusion with allograft. Spine J. 2004;4:184–91; discussion 191. 15016396 10.1016/j.spinee.2003.05.001

[21] Sugawara T Itoh Y Hirano Y. Long term outcome and adjacent disc degeneration after anterior cervical discectomy and fusion with titanium cylindrical cages. Acta Neurochir (Wien). 2009;15:303–9. discussion 309. 10.1007/s00701-009-0217-5 19262984

[22] Fujibayashi S Neo M Nakamura T. Stand-alone interbody cage versus anterior cervical plate for treatment of cervical disc herniation: sequential changes in cage subsidence. J Clin Neurosci. 2008;15:1017–22. 18653347 10.1016/j.jocn.2007.05.011

[23] Tabaraee E Ahn J Bohl DD. Comparison of surgical outcomes, narcotics utilization, and costs after an anterior cervical discectomy and fusion: stand-alone cage versus anterior plating. Clin Spine Surg. 2017;30:E1201–5. 29049131 10.1097/BSD.0000000000000341

[24] Yamagata T Takami T Uda T. Outcomes of contemporary use of rectangular titanium stand-alone cages in anterior cervical discectomy and fusion: cage subsidence and cervical alignment. J Clin Neurosci. 2012;19:1673–8. 23084624 10.1016/j.jocn.2011.11.043

[25] Kast E Derakhshani S Bothmann M. Subsidence after anterior cervical inter-body fusion. A randomized prospective clinical trial. Neurosurg Rev. 2009;32:207–14; discussion 214. 18797946 10.1007/s10143-008-0168-y

